# A Case of an Acquired Factor V Inhibitor Triggered by Levofloxacin and Ceftazidime and Effectively Treated With Clinical Pharmacist Assistance

**DOI:** 10.1002/ccr3.71129

**Published:** 2025-10-08

**Authors:** Chunjuan Zhao, Weibu Chen, Xueyan Chen

**Affiliations:** ^1^ Laboratory Department, Shenzhen People's Hospital, The First Affiliated Hospital Southern University of Science and Technology Shenzhen P.R. China; ^2^ Clinical Laboratory, Department of Clinical Laboratory Shenzhen Longhua District People's Hospital Shenzhen P.R. China

**Keywords:** acquired factor V inhibitors, asymptomatic, clinical pharmacist, coagulopathy, *self‐recovery*

## Abstract

Acquired factor V inhibitor is a rare and clinically challenging condition that can manifest across a spectrum ranging from asymptomatic laboratory abnormalities to life‐threatening hemorrhagic episodes. This case reports a patient with antibiotic‐associated AFVI, who presents asymptomatically and may be managed conservatively with close monitoring and clinical pharmacist guidance.

## 1 Introduction

Rare factor V (FV) inhibitors can develop at any age, and clinical symptoms display significant variability [1]. The clinical phenotype among patients with acquired FV inhibitors (AFVI) is diverse, ranging from asymptomatic presentations to thrombosis‐related symptoms, and may even result in life‐threatening hemorrhage [2]. Because of their atypical symptoms, most patients frequently present to non‐hematology departments, thereby adding to the complexity of diagnosis. In addition, factor V inhibitors can occasionally be misidentified as other inhibitors, presenting diagnostic challenges. This case report presents a patient with asymptomatic AFVI, potentially antibiotic‐induced, who recovered with clinical pharmacist assistance. Detailed clinical features, diagnostic evaluations, treatment course, and disease prognosis are presented, along with a review of pertinent literature.

## 2 Case History

A 92‐year‐old male patient has a long‐term indwelling gastric tube and a urinary catheter inserted due to Alzheimer's disease and the sequelae of cerebral infarction, receiving treatment at our hospital since 2021. On March 2, 2024, the patient underwent treatment with levofloxacin 0.5 g orally once daily for 20 consecutive days to treat a urinary tract infection caused by *Enterococcus faecalis* and *Proteus mirabilis* . During this period, ceftriaxone (2 g ivgtt qd) was administered for 15 consecutive days for anti‐infection purposes, as indicated by lung CT scans suggesting a complex lung infection. After a 28‐day treatment period, all symptoms of infection improved, resulting in the discontinuation of antibiotics.

### 2.1 Differential Diagnosis, Investigations, and Treatment

On the 15th day (March 17) of antibiotic therapy, we observed that the patient exhibited multiple rashes all over the body, with old ecchymosis on the left elbow. There were no other signs of bleeding, and the coagulation profile appeared normal, with prothrombin time (PT) of 13.2S, activated partial thromboplastin time (aPTT) of 34.4S, and thrombin time (TT) of 16.0S. Upon completion of the treatment on day 23 (March 25), no abnormalities were present in the coagulation profile, and there was no evidence of active bleeding. Seventeen days after treatment completion (April 11), the patient's left elbow ecchymosis had not fully resolved. Re‐examination revealed an abnormal coagulation profile with PT of 53.4 s, aPTT of > 180 s, and TT of 17.1S. The patient received daily supplementation of 400 mL of homotypic plasma on the same day for two consecutive days, but the coagulation function test showed no significant improvement. The physical examination did not reveal any bleeding in the patient. The patient had no known history of spontaneous bleeding or autoimmune disease, nor did they have any family members with such conditions.

**FIGURE 1:**
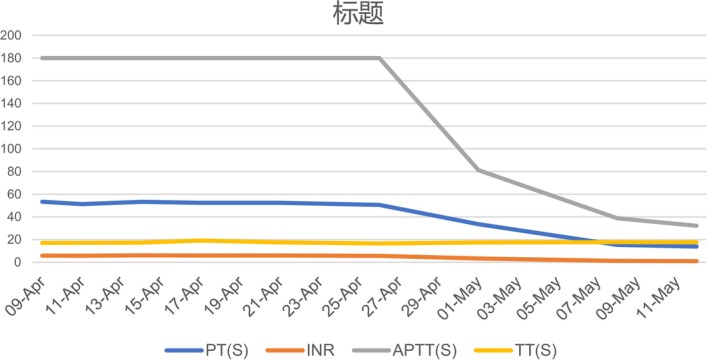
Trends in PT and APTT changes following discontinuation of plasma infusion/antibiotics. APTT, activated partial thromboplastin time; INR, international normalized ratio; PT, prothrombin time; TT, thrombin time.

**FIGURE 2:**
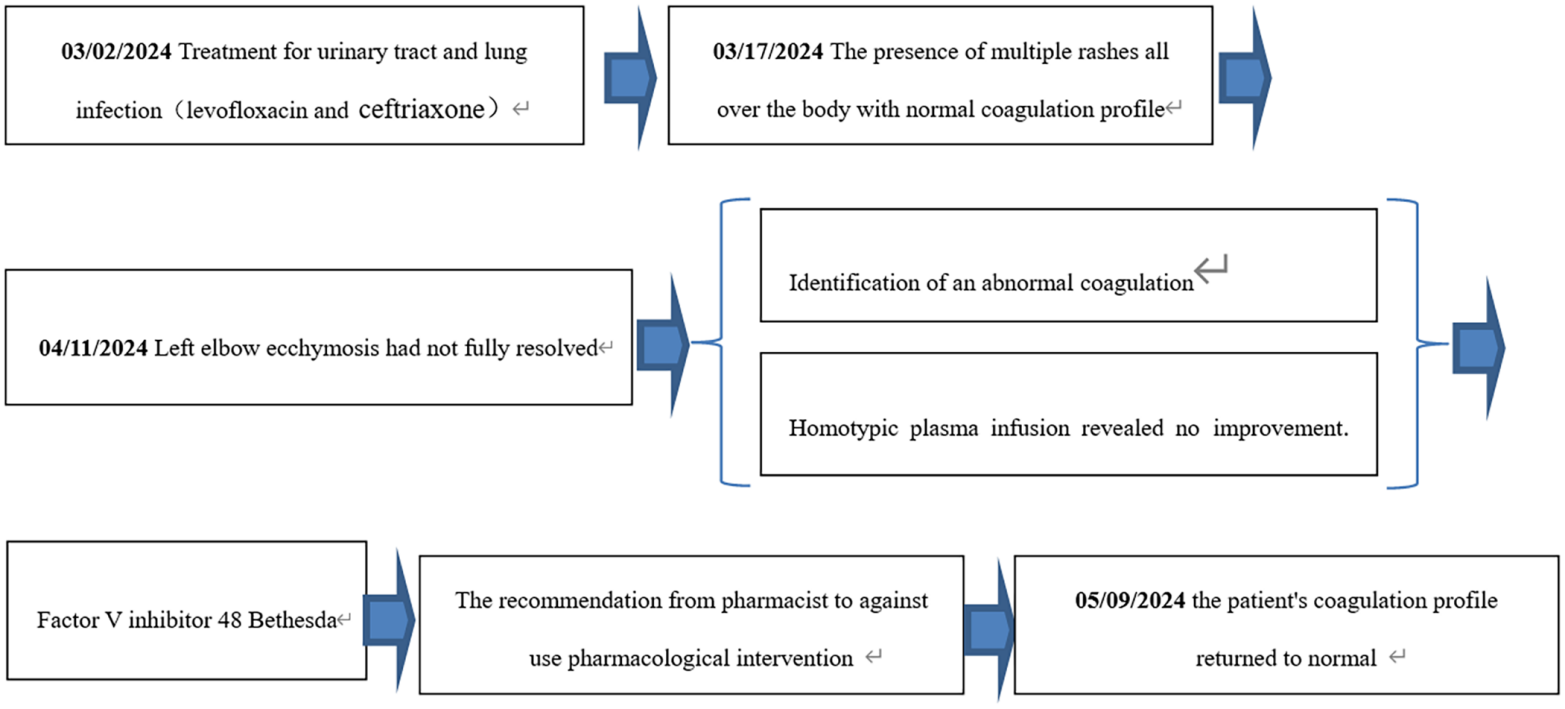
The chronological progression of primary treatment modalities and their effectiveness throughout the clinical process.

Therefore, following the hematologists' recommendation, the patient underwent a comprehensive coagulation profile test. The coagulation tests showed reduced activities of coagulation factors. The detailed examination results were as follows (Table 1): von Willebrand factor (vWF) antigen 210.0%, factor V activity < 5.8%, factor II activity 33.1%, factor VII activity 10.2%, factor IX activity 8.3%, factor X 27%, and factor XI activity 99.2%; after a 16‐fold dilution, coagulation factor activity returned to normal, revealing factor VII activity at 163.2% and factor IX activity at 132.8%; lupus‐like anticoagulants were not detectable due to interference from inhibitors; the activity of factors V, II, and X was still low when we used diluted patient's plasma samples; the addition of normal plasma in equal amounts to the mixing tests did not correct prolonged PT and aPTT; the FVi titers were 48 Bethesda units (normal range < 0.6 Bethesda), suggesting that the decreased activity of factor II and X in patients was due to a high titer Fvi; inhibitor interference resulted in lupus‐like anticoagulant undetectability. The diagnosis of AFVI was precise. For patients diagnosed with AFVI who are asymptomatic, treatment is generally considered unnecessary. Considering the patient's advanced age and the absence of documented bleeding during hospitalization, the clinical pharmacist advised against using steroids, rituximab, or any other drug regimens on April 9. Instead, the recommendation was to discontinue the six‐day homotypic plasma infusion and implement continuous monitoring along with periodic assessments of the coagulation profile. Closely monitoring the coagulation profile in the subsequent month demonstrated that the patient's parameters gradually normalized (Figure 1). The patient's successful therapeutic outcome can be attributed to the clinical pharmacist's involvement in formulating the drug treatment plan (Figure 2).

**TABLE 1:** Laboratory findings at the time of onset.

Test items	Result (normal range)
WBC (×10^9^/L)	4.6 (3.5–9.5)
RBC (×10^12^/L)	4.71 (4.3–5.8)
Hb (g/L)	145 (130–175)
Platelets (×10^9^/L)	169 (125–350)
PT (s)	53.4 (9.9–12.3)
PT (s) (mixing test)	46.9
INR	5.87
aPTT (s)	> 180 (20–40)
APTT (s) (mixing test)	146.9
Fibrinogen (g/L)	4.8 (2.0–4.0)
TT (s)	17.1 (14–21)
D‐Dimer (μg/mL)	1.52 (0.01–0.5)
Factor V (%)	< 5.8 (50–160)
Factor V (%) (16‐fold‐dilution)	< 5.8
Factor II (%)	33.1 (79–131)
Factor II (%) (16‐fold‐dilution)	40.2
Factor VIII (%)	10.2 (60–150)
Factor VIII (%) (16‐fold‐dilution)	163.2
Factor IX (%)	8.3 (67.6–128.5)
Factor IX (%) (16‐fold‐dilution)	132.8
Factor X (%)	27 (50–150)
Factor X (%) (16‐fold‐dilution)	35
Factor XI (%)	8.3 (60–150)
Factor XI (%) (16‐fold‐dilution)	132
Factor V inhibitor	48 Bethesda (< 0.6 Bethesda)
Lupus anticoagulant	Not determined (< 0.6 Bethesda)

*Note:* The mixing tests did not correct prolonged PT and aPTT. Following a 16‐fold dilution, the activities of factors VIII, IX, and XI normalized, while those of factors V, II, and X did not recover. Inhibitor interference resulted in lupus‐like anticoagulant undetectability.

## 3 Discussion

Before AFVI in this long‐term hospitalized elderly patient, there was no change in the diagnosis or treatment plan, aside from the use of suspicious drugs and repeated urinary tract infections. The coagulation profile was normal both when ecchymosis occurred and on the day of drug withdrawal. Based on the onset timeline, PT and aPTT were notably extended 17 days after the patient stopped antibiotics, gradually improved 41 days post‐medication discontinuation, and ultimately returned to normal levels 52 days post‐medication withdrawal, suggesting that the development of AFVI is associated with both antibiotics and infection. Our findings align with previous reports [3, 4, 5], indicating that all elderly patients developed AFVI following the administration of β‐lactams and quinolones due to systemic illness. In one case [5], the patient experienced a bleeding event; the FV inhibitor level normalized within approximately 4 months. Another case [3] involved an embolic event, where the FV inhibitor level normalized within a week of drug withdrawal without requiring additional drug treatment. In the final instance [4], upon confirmation of AFVI caused by cefotaxime, the clinician halted the administration of cefotaxime and administered prednisone for symptomatic treatment; ultimately, the patient succumbed to a non‐AFVI‐related cause of death.

Identifying any pharmaceutical factors, such as antibiotics, valproic acid, tacrolimus [6, 7], and other substances that could potentially stimulate the production of factor V inhibitors is crucial. Determining whether a specific drug is the causative agent is often challenging. Moreover, if an FVi emerges shortly after starting a new medication, it becomes crucial to discontinue its usage [8].

Factor V (FV) is in the coagulation cascade's common pathway; thus, FV inhibitors typically present with prolonged PT and aPTT [8]. TT is generally within the normal range; however, exposure to bovine thrombin may prolong the time [1]. FV is a coagulation factor with a distinctive structure consisting of heavy and light chains linked together by a connecting region. The precise mechanism underlying FV autoantibody formation remains incompletely understood, and a study revealed the presence of multiple binding sites for FV inhibitors. The study showed that patients with bleeding symptoms had inhibitors binding to the C2 domain of the light chain, ultimately interfering with the interaction between FV and the phospholipid membrane [9]. The inability of light chains to bind to phospholipids led to a significant decrease in affinity for FV and factor Xa, thereby diminishing the efficiency of prothrombin conversion to thrombin and being associated with bleeding [9]. FV inhibitors can exhibit lupus anticoagulant (LA) activity and notably suppress multiple coagulation factors. In this scenario, FV activity often experiences disproportionate reduction alongside other affected coagulation factors, showing only minimal improvement upon dilution compared to other factors. In this case, the FV activity level fell below 5.8%. After a 16‐fold dilution with normal plasma, all factors except FV reverted to normal levels. In comparison, FV activity persisted at less than 5.8%.

Infection is the most prevalent cause of AFVI, while antibiotics are the second major contributor. Beta‐lactam antibiotics are the most commonly used antibiotics [1]. Numerous related antibiotics are known to interact with phospholipids in the bacterial cell membrane that they target. Many theories have been proposed regarding the formation of antibiotic‐related AFVI. During immune enhancement states like infection, tumors, or autoimmunity, the immune system may abnormally produce antibodies against these antibodies, which may share some homology with the C2 region of the light chain of coagulation factors [9]. Stress‐mediated immune regulation may contribute to the generation of antibodies that can hinder the interaction of factor V with a compromised phospholipid membrane [10].

The primary treatment of AFVI consists of symptomatic management during acute hemorrhage and elimination of Factor V inhibitor [11]. Plasma exchange (PE), platelet transfusion, and fresh frozen plasma (FFP) are the preferred treatments for acute bleeding. Factor V exists in plasma (80%) and platelets (20%), and inhibitors do not readily neutralize the presence of coagulation factor V in platelets. Hence, platelet transfusion can be a treatment option for bleeding patients. In theory, factor V concentrate should be the optimal treatment choice; unfortunately, such a product remains unavailable. Immune suppression is indicated for eliminating factor V inhibitors, primarily utilizing prednisone, cyclophosphamide, and rituximab. Rituximab is particularly beneficial for patients with acquired coagulation factor V inhibitors who are unresponsive to alternative treatments.

Asymptomatic patients with AFVI generally do not require treatment. Based on the research findings [2], considering the patient's advanced age and the absence of documented bleeding during hospitalization, the clinical pharmacist advised against using steroids, rituximab, or any other drug regimens. Instead, the recommendation was to implement continuous monitoring along with periodic assessments of the coagulation profile. After 51 days without additional medical intervention, the patient's coagulation profile returned to normal, resulting in a satisfactory outcome.

## 4 Conclusion

Antibiotic‐associated AFVI may present asymptomatically and can be effectively managed through conservative treatment that incorporates close clinical monitoring and pharmacist guidance. This case demonstrates that a clinical pharmacist effectively leveraged his expertise to develop an optimal drug treatment plan for an asymptomatic AFVI patient with an antibiotic‐induced condition, ultimately facilitating the patient's recovery and discharge.

## Author Contributions

**Chunjuan Zhao:** conceptualization, data curation, resources, software. **Weibu Chen:** conceptualization, formal analysis, funding acquisition, resources, software, supervision, validation, writing – original draft. **Xueyan Chen:** conceptualization, methodology, supervision, writing – review and editing.

## Ethics Statement

The authors have nothing to report.

## Consent

Written informed consent was obtained from the patient for publication of this case report.

## Conflicts of Interest

The authors declare no conflicts of interest.

## Data Availability

Data sharing is not applicable to this article, as no datasets were produced or analyzed during this study.
